# Modifications of the metabolic pathways of lipid and triacylglycerol production in microalgae

**DOI:** 10.1186/1475-2859-10-91

**Published:** 2011-11-02

**Authors:** Wei-Luen Yu, William Ansari, Nathan G Schoepp, Michael J Hannon, Stephen P Mayfield, Michael D Burkart

**Affiliations:** 1 Department of Chemistry & Biochemistry, University of California San Diego, 9500 Gilman Drive, La Jolla, CA 92093, USA; 2 Division of Molecular Biology, University of California San Diego, 9500 Gilman Drive, La Jolla, CA 92093, USA; 3 The San Diego Center for Algae Biotechnology, University of California San Diego, 9500 Gilman Drive, La Jolla, CA 92093, USA

## Abstract

Microalgae have presented themselves as a strong candidate to replace diminishing oil reserves as a source of lipids for biofuels. Here we describe successful modifications of terrestrial plant lipid content which increase overall lipid production or shift the balance of lipid production towards lipid varieties more useful for biofuel production. Our discussion ranges from the biosynthetic pathways and rate limiting steps of triacylglycerol formation to enzymes required for the formation of triacylglycerol containing exotic lipids. Secondarily, we discuss techniques for genetic engineering and modification of various microalgae which can be combined with insights gained from research in higher plants to aid in the creation of production strains of microalgae.

## Introduction

In the past decade, the price of crude oil has ranged from 20 dollars a barrel to nearly 170 dollars a barrel. The volatile price, expected depletion and increase in atmospheric greenhouse gases due to oil combustion provide impetus to develop alternative energy sources. Biofuels have served as sources of energy from the beginning of human history, but the start of the industrial revolution led to a reliance on fossil energy due to its prevalence and high energy yields compared to the majority of bioenergy [1]. Establishing energy independence in coordination with the increasing costs for liquid fuels have renewed interest by the government, industry and academia in renewable liquid fuels to replace petroleum.

Biofuels can be solids, liquids or gasses so long as they are derived directly from biological sources. The most common solid biofuel is lignified cellulose (wood) that can be burned for energy. Liquid and gaseous biofuels generally require more refining, and include bioethanol, biodiesel, and engine-combustible hydrocarbons as well as methane from anaerobic digestion. The aforementioned liquid biofuels offer significant potential to augment or replace petroleum gasoline for transportation purposes. Currently ethanol dominates the biofuel market and may be produced by a variety of methods, primarily heterotrophic fermentation of sugars purified from biomass feedstocks [2]. Biodiesel, and other hydrotreated biofuels, are derived mainly from vegetable oil feedstocks (lipids) [3].

The lipids used for biofuels have important physiological roles in plants, including energy storage, structural support as membranes, and intercellular signaling [4]. Storage lipids differ from both structural and signaling lipids in that they are mainly composed of glycerol esters of fatty acids, also known as triacylglycerol (TAG). These lipids are generally stored in a compartment specialized for lipid storage, the lipid body. This compartment is found in most oleaginous plant cells, and is used to store a variety of TAG molecules depending on the species [5]. Vascular plants store large amounts of lipids in seeds, and provide energy for growth during germination. The lipid content, and fatty acid composition of oilseeds varies. Environmental changes or human manipulation, such as breeding or genetic engineering have been used to change lipid content and composition [6]. Although less common, some species like *Simmondsia chinensis *accumulate storage lipids as waxes rather than as TAG. Regardless of the final storage type, *de novo *fatty acid biosynthesis in plants occurs exclusively in the stroma of plastids, whereas, with the exception of plastidial desaturation and a few complex lipid biosyntheses, most modifications of fatty acyl residues and TAG synthesis from acyl chains are localized in the lumen of the endoplasmic reticulum (ER) [6]. In addition to TAGs, plants also contain membrane lipids. These, unlike TAGs, remain highly conserved in both identity and quantity to maintain normal plant physiology.

Ethanol and biodiesel is primarily derived from plant sources, often food crops, because the established scale of food crops made them a convenient source of biomass necessary to produce biofuel on a commercial scale. However, an increasing demand for biofuel feedstocks has negatively impacted food markets, and raised a global "food vs. fuel" controversy. Furthermore, the land and fresh water requirements for growing crops, and the long growth-to-harvest periods limit the expansion of plant based biofuel industries to the amount of arable land. In contrast, unicellular algae require smaller amounts of land that does not need to be arable, have faster growing cycles, contain a higher percentage of oil, and have been proposed to be a better solution to the food vs. fuel debate. Therefore, significant attention has been focused on algae as a next generation feedstock for biofuel production [7]. It has been proposed that a fuel only based approach to biodiesel production from algae is unlikely to be feasible with current yields based on economic modeling of production facilities. As a result, attention must be paid to genetic manipulations in order to harness the ability of algae to make high quality fuel, but also potentially to serve as a factory for the production of other value added products such as protein therapeutics [8,9]. In light of this and studies on selection pressure for photosynthetic efficiency in native vs. bioreactor environments, it seems genetic modification is likely to provide the key to unlocking the feasibility of algal production strains [10].

Several research papers and reviews have been published presenting the recent progress in plant lipid biosynthesis and related industrial applications [4,11-14]. In this review, we discuss lipid biosynthesis and regulation in plants and algae; the state of genetic manipulation in plants to modify lipid biosynthesis; and the possible impacts of manipulation on biodiesel production from algae and future studies.

### Biosynthesis of Triacylglycerol (TAG) in Plants

**Figure 1:**
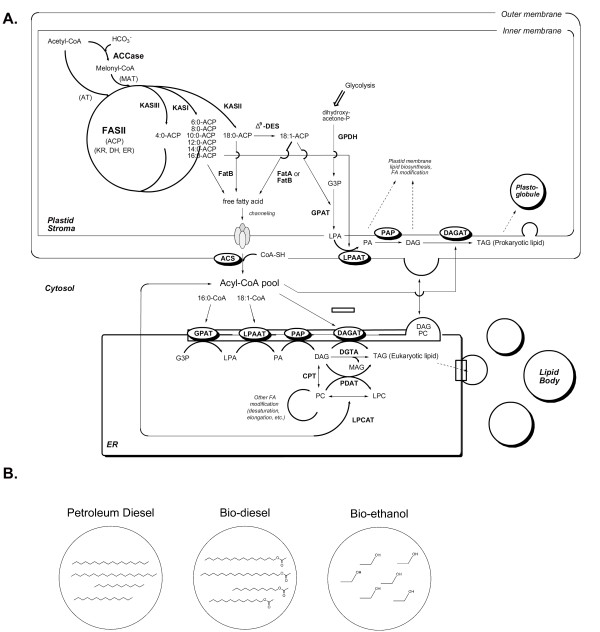
**The general scheme of plant lipid biosynthetic pathway and representative chemical structures of petroleum diesel, biodiesel, and ethanol**. AT = acetyltransferase, MAT = malonyl-CoA acetyltransferase, ACP = acyl carrier protein, KAS = ketoacyl synthase, FAS = fatty acid biosynthesis, KR = ketoreductase, DH = dehydratase, ER = enoyl reductase, GPDH = glycerol-3-phosphate dehydrogenase, GPAT = glycerol-3-phosphate acyltransferase, LPAAT = lysophosphatidic acid acyltransferase, PAP = phosphatidic acid phosphatase, DAGAT = diacylglycerol acyltransferase, ACS = acetyl-CoA synthetase, DGTA = diacylglyceryl hydroxymethyltrimethyl-β-alanine, CPT = carnitine palmitoyl transferase, PDAT = phospholipid diacylglycerol acyltransferase, LPCAT = lysophosphatidylcholine acyltransferase.

A general scheme of plant TAG biosynthesis is broadly discussed in the textbook "Plant Lipid Biosynthesis: Fundamentals and Agricultural Application" and other review articles, as shown in Figure 1. The TAG pathway begins with the basic fatty acid precursor, acetyl-CoA, and continues through fatty acid biosynthesis, complex lipid assembly, and saturated fatty acid modification, until finally reaching TAG formation and storage [15].

The fatty acid synthase (FAS) complex residing in plant chroloplasts is a major player in *de novo *fatty acid synthesis [16]. Completion of *de novo *fatty acid synthesis is accomplished in one of three ways [4,6]. Either the newly synthesized fatty acid is hydrolysed by a thioesterase, further modified by desaturases, or directly transferred to complex lipid formation ('prokaryotic lipid') using plastid acyltransferases. After release from plastids, free fatty acids are exported to the cytosol by an unknown mechanism and converted to acyl-CoA esters by an acyl-CoA synthetase located in the outer envelope of the plastid [17,18]. The cytosolic acyl-CoA esters are then transferred to the ER for further elongation, modification, or participation in the synthesis of membrane lipids or storage TAGs ('eukaryotic lipid').

Triacylglycerols (TAGs) are commonly found as storage fats or oils and are described as neutral or non-polar lipids, differentiating them from polar membrane lipids. TAGs consist of three FA chains esterified via the hydroxyl groups of a glycerol backbone. Biosynthesis of TAGs occurs in the plastids, mitochondria, and endomembrane system. Although the substrates are commutable, each compartment of the plant cell has an independent TAG synthesis pathway. The Kennedy pathway is well understood and one of the most straightforward TAG biosynthesis pathways; it consists of stepwise acylation, adding to each hydroxyl group of glycerol beginning with glycerol-3-phosphate [19]. Lipid bodies are single-layer, membrane-wrapped, protein embedded organelles 0.2-2.5 μm in diameter and are in the cytoplasm of most, if not all, plant cells [4,5]. It is generally believed that plant lipid bodies are not only a cellular lipid reservoir, but also provide an effective energy battery during seed germination. Plastoglobules are lipid bodies found within plastids that contain TAG, isoprenoid-derived metabolites, as well as proteins [20].

Palmitate (16:0) and stearate (18:0) are the major products of plastid FAS. However, the major fatty acids of plants are the C18 compounds, oleate (18:1Δ9), linoleate (18:2Δ9, 12) and α-linolenate (18:3Δ9, 12, 15). Together, these three fatty acids represent over 85% of total membrane acids, and over 80% of economically important storage oils. There are other various fatty acids which contain longer carbon-chains, unsaturated double bonds, hydroxyl groups, and other modifications within the plant fatty acid repertoire [21].

Fatty acid modification during plant lipid biosynthesis is crucial for generating the fatty acid repertoire found in plants. Fatty acids with 20 or more carbon atoms are called very-long-chain fatty acids (VLCFAs). In plants, VLCFAs are ubiquitous in leaf surfaces as wax, and in cuticle components which play an important role against xenobiotics. In *Simmondsia chinensis *seed, VLCFA is the major component of energy storage in the form of liquid wax consisting of chains 36 to 46 carbon atoms in length [11,13]. The VLCFAs are precursors of very-long-chain polyunsaturated fatty acids (VLC-PUFAs) which are important in human nutrition and health [22]. However, none of these VLC-PUFAs is normally produced in higher plants. In lower eukaryotes such as mosses, fungi, and algae, VLC-PUFAs are synthesized to confer flexibility, fluidity, and selective permeability to cellular membranes in stringent environments [23].

### Biosynthesis of Triacylglycerol (TAG) in Algae

Algae are a diverse group of organisms which includes prokaryotes and eukaryotes in the form of single cells, colonized cells, and multicellular plants. Algae are typically distinguished from other classes of organisms by their ability to fix carbon, and utilize solar energy. Algae reside in a variety of ecosystems including marine and freshwater environments, desert sands, hot springs, and even snow and ice. To survive in these environments, algae produce a myriad of lipids. These include structural lipids for cellular membranes, as well as lipids for nutrient storage [24,25]. The two oil crises during the 1970's spurred a vigorous search for alternative energy sources, as people began to address their growing energy problem. From 1978 to 1996, the U.S. Department of Energy's Office of Fuels Development developed the Aquatic Species Program (ASP) with a goal of developing renewable transportation fuels from algae. During this program, systematic and fuel-directed algal oil research evaluated the potential of algal oil as an energy source. Although the program was terminated in 1996, the preliminary results of the pioneering studies provide a direction for later exploration in this field [26].

Over almost two decades of the ASP program, thousands of algae strains were isolated and screened for their lipid and fatty acid content. These data were combined with previous sporadic results, and some generalizations of lipid content in different algae categories were formed [26-29]. For example, diatoms are among the most common and widely distributed groups of algae. They store energy primarily in the form of lipids (TAGs) and the average lipid content of oleaginous diatoms is 22.7% dry-cell-weight (DCW) under normal growth conditions; with that number rising to 44.6% DCW when cultured under stress conditions [30]. However, the slower growth rate caused by nutrient deficiency, along with the increasing cost of silicate containing culture media hampers the usage of diatoms as a robust biofuel feedstock.

Green algae, often referred to as chlorophytes, are highly abundant and are estimated to number as many as 8,000 species. They are the most diverse group of algae, and include unicellular, colonial, coccoid, filamentous, and multicellular forms growing in a variety of habitats. Green algae are believed to share a common ancestor with higher plants, carrying the same photosynthetic pigments and having similar metabolic mechanisms. Generally, these algae use starch as their primary storage vehicle, however, in some strains large quantities of TAG accumulate under specific growing conditions. Oleaginous green algae contain an average total lipid content of 25.5% DCW, which can be raised to 55.2% DCW when the algae are grown under stress conditions or heterotrophically [30,31]. *Chlamydomonas reinhardtii *has been treated as a model organism for photosynthesis, and as a result has been studied extensively, because of its giant chloroplast and ability to control sexual reproduction, allowing detailed genetic analysis [32]. Indeed, *Chlamydomonas *was also the first alga to be genetically transformed and a draft sequence of the whole genome has recently been determined [33]. Although it does not typically accumulate lipids under ideal conditions, metabolic engineering can be used to transform this alga into an oleaginous factory [34].

Algal lipid metabolism from *de novo *fatty acid biosynthesis to the formation of complex glycerolipids is similar to that of the plant cells. Higher plants have differentiated organs, each of which performs specific physiological functions, and contains specific biochemical pathways. Similarly to higher plants, algae process TAG into lipid droplets which are coated in a large number of proteins. Most of these are typical members of vesicular transport and signaling pathways such as RabGTPases, but a proteomics approach to algal lipid bodies has identified a protein called major lipid droplet protein (MLDP) which affecs the size of lipid droplets and may present a target for immunofluorescence imaging of algal lipid content [35]. Algae species, especially microalgae, have a general biochemical composition of 30-50% DCW proteins, 20-40% DCW carbohydrates and 8-15% DCW lipids under optimal growth condition[36]. Most of the algal lipids are glycerinated membrane lipids, with minor contributions to overall lipid content from TAG, wax esters, hydrocarbons, sterols, and prenyl derivatives [30,36]. Under unfavorable growing conditions many algae shift their metabolic pathways toward the biosynthesis of storage lipids or polysaccharides. TAG accumulation in response to environmental stress likely occurs as a means of providing an energy deposit that can be readily catabolized in response to a more favorable environment to allow rapid growth [27]. Nutrients, temperature, light, salinity and growing phase have been shown to influence the flux of algal cellular metabolism [37].

Since many of the algal lipid metabolism studies on environmental changes have been carried out in batch cultures, there is a lack of systematic, multi-factor monitored studies. This decreases the practicability of applying previous findings to large-scale algal cultures. During the years of Aquatic Species Program, a 'silver bullet' was sought; a single species which could produce high levels of storage lipids without growth rate alteration. To maximize lipid production and growth efficiency for industrial scale culture, experiments with recombinant genetics and complex culture conditions (multi-stage cultures, timed nutrient limitations) may be required.

### Engineering of Lipid Biosynthesis for the Production of Biofuels

#### 1. Advantages of Biodiesel

Petroleum diesel or petrodiesel is a mixture of saturated and aromatic hydrocarbons with 10-15 carbon atoms and is ignited in high-compression diesel engines. Most plant oils (TAGs) are too viscous to use in modern diesel engines, and eventually lead to engine failure caused by incomplete combustion. Biodiesel is mono-alkyl (usually methyl) esters (fatty acid methyl ester, or FAME) made by the transesterification of TAGs from vegetable oils or animal fats, and has a similar viscosity to petrodiesel [38]. There are several advantages in addition to carbon neutrality when using biodiesel as a liquid fuel source. The cetane number, a measure of the delay between compression and ignition, can be higher for biodiesel than regular grade petrodiesel. This reflects the quality of the fuel and a higher number is associated with shorter delays in ignition, resulting in more complete combustion. Burning biodiesel produces less carbon monoxide, particulate matter, sulfur, and aromatic compounds than burning petrodiesel. Furthermore, it has a higher flashpoint, allowing safer handling and storage and greater lubricity for engines than other fuels. It is made from renewable biomass and is biodegradable and "friendlier" to the environment than crude petroleum when fuel leakages do occur. Currently only two major renewable liquid fuels are produced in large quantities, bio-ethanol and biodiesel. Biodiesel has 25% higher energy content per volume, and requires much less energy input in production than bio-ethanol, as no distillation step is necessary. Additionally, ethanol has been shown to corrode pipelines, likely shortening their lifetimes [39].

Despite the many advantages, and increasing market share of biodiesel, there are limitations hindering its complete replacement of petrodiesel [38]. Negative biodiesel characteristics include poor cold-temperature properties, namely the tendency to solidify or gel, which can lead to fuel starvation and engine failure. The presence of polyunsaturated fatty acids in biodiesel also makes it susceptible to oxidation by atmospheric oxygen or hydrolytic degradation by water, which decrease the stability of biodiesel during long-term storage. In addition, the emissions from biodiesel contain a higher concentration of nitrogen oxide (NO_x_) than do petrodiesel emissions, limiting its usage in areas under strict air quality standards. One of biodiesel's biggest limitations is cost and supply. As mentioned above, the use of oil crops for biodiesel production has already increased the cost of these commodities, and raised the 'food vs. fuel' debate. Although the oil supply problem may be relieved by switching from food plant to non-food plant feedstocks such as algae, the higher production costs of algal oil along with the lack of successful industry examples to date further hinders industry-scale adoption of algae-derived biodiesel.

The four major sources of plant oil today are oil palm, soybean, rapeseed, and sunflower, which together account for approximately 79% of the world's total production. Within these oils, palmitate (16:0), stearate (18:0), oleate (18:1Δ9), linoleate (18:2Δ9, 12), and α-linolenate (18:3Δ9, 12, 15) are the five main fatty acid components [14]. Unusual fatty acids produced by specific plant species contain unique functional groups giving them selective usages in industry [4]. The fatty acid composition determines the physical and chemical properties of the oil and its economic value. Traditionally, simple methods like blending or partial hydrogenation were applied to produce oils for specific applications. As the accumulating knowledge of plant lipid biosynthesis has been coupled with the development of advanced genetic technologies, various metabolic engineering methods have been performed to modify the fatty acid and lipid composition of several oleaginous plants [40-42].

#### 2. Increasing Oil Content

Increasing oil content could be a straight-forward method to lower the high cost of biodiesel production, and may be applicable through genetic manipulation of lipid biosynthetic pathways. Table 1 shows an outline of genetic manipulations that have been performed in higher plants and the resulting changes in fatty acid composition and content. It has been proposed that lipid biosynthesis may be controlled by the availability of fatty acids, and that the production of fatty acids is regulated by acetyl CoA carboxylase (ACCase) [43,44]. Increasing the activity of ACCase may push excess substrate, malonyl-CoA, into the lipid biosynthesis pathway. Substantially increasing plastidial ACCase activity may prove quite complex due to the multigene-encoded enzyme complex and its post-translational regulation [45]. A successful example has been achieved by expressing a cytosolic version of the enzyme targeted to the rapeseed chloroplast [46]. This manipulation resulted in a higher ACCase activity and consequently a 5% increase in seed oil content, a relatively modest increase.

**Table 1:** A list of genetic modifications to higher plants and their resulting changes in fatty acid content

Modification	Organism	Result	Reference
Expression of a cytosolic variant of endogenous ACCase	*Brassica napus*	5% increase in seed oil content	[40]

Expression of KASIII from *Spinacia oleracea*	*Brassica napus*	Increased palmitic acid proportion, decreased total fatty acids 5-10%	[41]

*Saccharomyces cerevisiae *G3p dehydrogenase (gpd1)expression	*Brassica napus*	40% increase in seed oil content	[43]

*Carthamus tinctorius *G3p acyltransferase (GPAT) expression	*Arabidopsis thaliana*	10-21% increase in seed oil content	[45]

*Saccharomyces cerevisiae *sn-2 acyltransferase (SLC1-1) expression	*Brassica napus*	53-121% increase in erucic acid content	[47]

*Arabidopsis thaliana *diacylglycerol acyltransferase (DGAT1) expression	*Brassica napus*	Increases in oil content and seed weight	[48]

Down regulation of FAD2 desaturase and FatB hydrolase	*Glycine max*	85% increase in oleic acid levels	[53-55]

Expression of *Coriandrum sativum *Δ4palmitoyl ACP desaturase	*Nicotiana tabacum*	< 10% of total fatty acid became palmitoleic acid	[56]

Expression of *Thunbergia alata *Δ6 ACP desaturase	*Arabidopsis thaliana*	< 10% of total fatty acid became palmitoleic acid	[57]

Expression of *Umbellularia californica *lauryl-ACP thioesterase	*Arabidopsis thaliana*	24% of total fatty acid converted to laurate	[66]

Expression of *Umbellularia californica *lauryl-ACP thioesterase	*Brassica napus*	58% of total fatty acid converted to laurate	[67]

Expression of *Cuphea hookeriana *FatB1 thioesterase	*Brassica napus*	Fatty acid content changed to 11% caprylate and 27% caprate	[68]

Co-expression of *Cuphea hookeriana *FatB1 thioesterase and KAS (ketoacyl ACP synthase)	*Brassica napus*	30-40% increase in short chain fatty acid content over FatB1 expression only	[69]

Co-expression of *Cuphea hookeriana *FatB1 thioesterase and LPAAT from *Cocos nucifera*	*Brassica napus*	67% of total fatty acid content converted to laurate	[70]

Increasing malonyl-CoA substrate pools for de novo fatty acid biosynthesis resulted in only minor increases in seed oil yield. Fatty acid synthase has been suggested to be another rate-limiting regulator of lipid production and several studies have been performed where a single enzyme of the FAS complex is overexpressed. Heterologous overexpression of KAS III, the first condensing enzyme synthesizing 4C acyl chains, increased the proportion of palmitic acid (16:0) but decreased the total fatty acid content by 5-10% [47]. The accumulation of butyryl-ACP suggests that KAS I is the next rate-limiting enzyme. It seems unlikely that the up-regulation of any single enzyme will have a major positive effect on lipid biosynthetic flux. Multiple gene expression or activation of key regulators operating on the entire fatty acid biosynthetic pathway may have a more substantial effect on lipid production [48].

The second part of triacylglycerol biosynthesis is the Kennedy pathway, which depends on levels of glycerol-3-phosphate. Increasing the glycerol-3-phosphate levels in developing seeds by overexpression of a yeast gene encoding a cytosolic glycerol-3-phosphate dehydrogenase (*gpd1*) resulted in a substantial increase in seed oil content up to 40% in transgenic rape [49,50]. Other successful examples increasing plant oil levels have come by altering the acyltransferases of TAG biosynthesis. *Arabidopsis thaliana *has been transformed with a soluble safflower glycerol-3-phosphate acyltransferase (GPAT), where the plastidial targeting sequence was removed, and an *Escherichia coli *GPAT inserted. Seeds of both transgenic plants produced 10 to 21% more oil [51]. A yeast *sn*-2 acyltransferase gene (*SLC1-1*) was introduced into a high erucic acid (22:1Δ9)-containing *Brassica napus*. The resulting transgenic strain showed a substantial increase in seed oil content and an increase in the proportion of erucic acid [52]. The transgenic strain was later tested in the field, and exhibited a 53-121% increase in total erucic acid yield (weight/plot) [53]. Overexpression of the Arabidopsis *DGAT1 *gene in the wild-type strain led to increased seed oil deposition and average seed weight [54]. A functional DGAT homologue, the *DGAT2 *gene from the oleaginous fungus *Mortieralla rammanniana *was overexpressed in soybean, and resulted in small but significant increases in seed oil content in both greenhouse and field tests [55].

Together, these studies indicate that increased metabolic flux towards oil production may be achieved by manipulations targeted at later steps in the TAG biosynthetic pathway. A reasonable explanation is that the consequences of activating early biosynthetic steps may be slowed by later rate-limiting steps, and excess intermediate products may be utilized by other metabolic pathways sharing the same intermediates of TAG biosynthesis. Metabolic modeling networks that simulate flux of fatty acids through TAG biosynthetic pathways should play an important part in developing strategies for future genetic manipulation. Actual values of the engineering results need to be properly calculated for whole organisms and total production costs, not just the oil itself. For example, increasing oil content of soybean usually comes at the expense of the reduction of high-value protein content used for animal feed. Rigorous field testing is necessary to determine whether oil content increases are reflected in an increased oil yield per hectare per year. These tests must prove that strains with lipid content increases are economically viable compared to elite, high-yield commercial varieties.

#### 3. Changing the Fatty Acid Composition of Oil

Beyond base supply, biodiesel has other limitations hindering its market competitiveness. The fuel properties of biodiesel are closely related to its fatty acid composition. Altering the fatty acid profile, for example the carbon chain length and number of double bonds, can lead to a better-quality, inexpensive biodiesel. The presence of methyl ester with saturated acyl chain longer than C12 significantly increases the cloud point of the biodiesel, the temperature at which crystals form [56]. The methyl esters derived from poly-unsaturated fatty acids are prone to oxidation and the hydroperoxides formed will eventually polymerize and form insoluble sediments capable of interfering with engine performance [57]. Highly saturated and longer carbon chain esters have lower NO_x _emissions relative to shorter, less conjugated chains [58]. In addition, biodiesel ignition quality is adversely effected by an increase in the number of double bonds [38]. When requirements for biodiesel quality are viewed together, it is clear no single fatty acid methyl ester (FAME) could fulfill every parameter. However, a balance of different fatty acids containing higher amounts of mono-unsaturated fatty acids such as oleate (18:1Δ9), and fewer saturated and polyunsaturated fatty acids would yield a more reliable biodiesel [59].

##### Increasing the contents of monoenoic fatty acids

Most polyunsaturated fatty acids in storage lipids are derived from oleic acid by the catalysis of FAD2 (ω6) homologues. Therefore, down-regulation of the ER membrane-bound fatty acid desaturases should result in an increased percentage of oleic acid present, relative to total fatty acid content. Several experiments have successfully enhanced the oleate concentration in various oleaginous plants [60-62]. Down-regulating *FAD2 *and *FatB*, which hydrolyzes the saturated acyl-ACP, further increases oleic acid levels in transgenic soybean to over 85%, with saturated fatty acid levels at less than 6%. In addition to oleic acid, other unusual monoenoic fatty acids from plants have potential for biodiesel production. Introduction of a coriander Δ4 palmitoyl (16:0)-ACP desaturase, or a *Thunbergia *Δ6 palmitoyl-ACP desaturase into tobacco callus and *Arabidopsis *seed, respectively, resulted in less than a 10% accumulation of these non-native unusual fatty acids and their derivatives [63,64]. Similar experiments have been performed in *Arabidopsis *and *Brassica napus *where Δ9 palmitoyl-ACP desaturase from *Uncaria tomentosa *was introduced. Significant increases in palmitoleic acid (16:1Δ9) and its derivatives were found in both transgenic plants, although the proportion of palmitoleic acid to total fatty acid content was much lower than the original *Uncaria tomentosa *(80%) [65]. The reason for the low levels of unusual monoene production in non-native plants may be lack of corresponding ACP, ferredoxin, 3-ketoacyl-ACP synthase, thioesterase, and acyltransferase present in the original strains [64,66]. Since fatty acid desaturases are highly conserved in their structure and amino acid sequences, several chimeric enzymes have been generated and shown to have broader substrate specificity [67,68]. These engineered desaturases may be more effective when designing transgenic plants to produce large amounts of monoenoic fatty acids [69].

##### Engineering of fatty acid chain length

As mentioned previously, fatty acyl chain length is another important factor that influences the viscosity and cold flow properties of biodiesel [38]. Short- to medium-chain fatty acids (C8-C14) have lower viscosity and higher cloud points than common long-chain fatty acids (C16-C18). Although cold-flow properties are superior, cetane numbers are lower, and overall NO_x _emissions higher for shorter chain fatty acids. However, increasing their proportion in market-available biodiesel still leads to better quality, more competitive fuel in terms of combustion performance.

Commercial oils from palm kernel and coconut oil contain > 40% of total fatty acids in the form of lauric acid (12:0). Plants that accumulate short- to medium-chain (C8 to C14) fatty acids in seed oil contain chain-length-specific acyl-ACP thioesterases that cleave the corresponding fatty acids from the growing acyl-ACP of *de novo *fatty acid biosynthesis [70]. For example, *Umbellularia californica *and *Cuphea hookeriana *seeds accumulate up to 90% short- and medium-chain saturated fatty acids in triacylglycerols. The chain-length-specific acyl-ACP thioesterases were identified in both species as the cause of the unusual accumulation [71,72]. The expression of a lauryl-ACP thioesterase from *Umbellularia californica *in the seeds of non-laurate-accumulating plants, *Arabidopsis *and *Brassica napus *(rapeseed), resulted in laurate quantities as large as 24 and 58% of total seed fatty acids, respectively [73,74]. In another transgenic experiment, a medium-chain thioesterase, Ch FatB1 from *Cuphea hookeriana*, which produces 50% caprylate (8:0) and 25% caprate (10:0) in their total fatty acids, was introduced into rapeseed. The transgenic rapeseed was found to accumulate up to 11% caprylate, and 27% caprate [75]. The reasons for lower production of short-chain fatty acids in transgenic hosts compared to donor species were further investigated. A short-chain-fatty-acid-specific condensing enzyme (3-ketoacyl-ACP synthase, KAS) from *Cuphea hookeriana *was identified and co-expressed with Ch FatB1 in rapeseeds. All double-transgenic lines showed a 30-40% increase in the levels of short-chain fatty acids compared to the Ch FatB1 single-transgene rapeseeds [76]. Additionally, structural analysis of TAG from the plants containing inserted medium-chain acyl-ACP thioesterase revealed that laurate was only present at *sn*-1 and *sn*-3 positions [74]. The high specificity of lysophosphatidic acid acyltransferase (LPAAT) from the hosts prevented laurate from being incorporated at the *sn*-2 position of TAG. Co-expression of a laurate-specific coconut LPAAT into rapeseed containing the *Umbellularia californica *thioesterase resulted in further increases in laurate levels, up to 67% of the total fatty acid content [77]. Another lesson learned from the study of laurate-producing transgenic plants was the importance of enzymes for lauryl-CoA β-oxidation, malate dehydrogenase, and isocitrate lyase, all of which participate in the glyoxylate cycle for fatty acid carbon reutilization. These genes were induced with increasing levels of the lauric acid [78]. Obtaining significant amounts of short-chain fatty acids in TAG may require the engineering of multiple genes, including the short-chain-specific keto-synthase and thioesterase, as well as short-chain-specific acyltransferases, which assemble the novel fatty acids into TAG. Production of unusual fatty acids in transgenic hosts can induce antagonistic pathways reducing the effects of genetic manipulation, which must be addressed to maximize production efficiency.

Recently, direct use of low-molecular-weight TAG as fuel has been discussed and studied [59,79]. The lower cost of TAG fuels on the transesterfication and purification of FAMEs greatly enhances the market potential of such biodiesels. Seed oil containing 40% of caprylate (8:0) and 37% caprate (10:0) in total fatty acids from a mutant *Cuphea viscosissimal *had a coking index (a measure of engine carbon deposition) comparable to that of No. 2 diesel used by on road vehicles in the US, albeit with the problem of poor low-temperature viscosity [80,81]. Another interesting study involves the 1,2-diacyl-3-acetyl-*sn*-glycerols (ac-TAG) from the seeds of *Euonymus alatus *(Burning Bush). This acetyl TAG has a lower viscosity than common TAGs, and the potential to be used directly as biodiesel [59]. This specific acetyl DAGAT has been isolated from *Euonymus alatus*, and data on the oil properties of transgenic plants are much anticipated [82].

#### 4. Manipulation of Algal Lipid Metabolism Using Genetic Engineering

During the years of ASP (Aquatic Species Program), an extra-copy of the monomeric ACCase gene was introduced into the genome of the diatom *Cyclotella cryptica*, in an attempt to increase lipid accumulation in the transformed strains [83]. Unfortunately, a two to three-fold higher ACCase activity in the transformed algae did not result in any enhancement of lipid production [26]. A major reason very few positive engineering results have been achieved in algae lipid metabolism is the lack of a reliable nuclear transformation system like that used in higher plants. A more promising method of genetic engineering has been successfully established in the chloroplast of *Chlamydomonas reinhardtii *[84]. However, as the examples in vascular plants have shown, most of the critical enzymes controlling lipid biosynthesis and fatty acid modification reside in the cytoplasm.

Several transformation techniques have been developed to genetically engineer *C. reinhardtii *to express recombinant proteins from both the chloroplast and nuclear genomes. General transformation protocols such as electroporation, particle bombardment, silicon carbide whisker agitation, and even *Agrobacterium tumefaciens *have been shown to transform a number of diverse microalgae including both green and red algae, diatoms, and dinoflagellates [85-89]. Expression levels vary greatly depending on a number of factors including auto-attenuation of exogenous sequences, codon usage bias, GC content, and proteasome mediated degradation [90]. Improvements in nuclear expression of transgenes have been reported with combined codon usage optimization, endogenous 5'/3' UTRs, and inserting introns from endogenous genes [91,92]. Transformation and expression research in *C. reinhardtii *will likely translate to a better understanding of microalgae gene silencing mechanisms and therefore more effective means to prevent transgene silencing in a variety of microalgae species.

Transformation of the nuclear genome allows for inducible gene expression, targeting to subcellular compartments, and protein secretion [93]. Insertion typically occurs via non-homologous recombination, though homologous recombination is known to occur at a very low frequency [94]. Optimizing homologous recombination conditions should allow for the directed knockout of enzymes diverting carbon usage away from lipid production, or for the directed replacement of lipid synthesizing enzymes with more effective isozymes. High levels of transgene expression can be selected for by using antibiotic resistance genes in combination with transgenic constructs. Addition of the *ble *gene to a transgenic construct confers resistance to phleomycin and zeocin in a 1:1 drug:protein ratio and can be used to select for transformants with high expression levels [95].

Although the nuclear genome does not yet robustly support protein production on a scale viable for harvesting protein therapeutics such as antibodies, expression of cytosolic enzymes or signaling proteins which promote the production of storage lipids may reach high enough activity levels to significantly alter the overall lipid profile of the host microalgae. *C. reinhardtii *and the model organism diatom *Phaeodactylum tricornutum *are known to produce fatty acids under nitrogen starved conditions and deletion of sta6 (involved in starch biosynthesis) in *C. reinhardtii *significantly increased lipid production in response to nitrogen starvation [34,96]. Endogenous micro RNAs (miRNA) and RNAi machinery have been shown to function and knock down gene expression in *C. reinhardtii*, and furthermore selectable constructs for artificially knocking down gene expression using RNAi machinery have been developed, enabling a reverse genetics approach to probing gene function [97-99]. High-throughput screening by insertional mutagenesis could be followed up with an RNAi based approach to investigate pathways for regulators of stress response, which may yield a genetic mechanism to increase lipid yield while minimizing growth arrest in large scale cultures. RNAi of protein members of pathways involved in lipid catabolism such as lipase and proteins of the beta oxidation, glyoxylate, and gluconeogenesis may represent important modifications which could increase overall TAG content [100].

To date, there have been over 30 complete genome sequences of algae determined, with still more unpublished [30,101]. With this primary sequence data and the functional characterization of homologous plant genes in hand, we can more precisely determine the key regulators of algal lipid biosynthesis *in silico*. Work has already started in this field, including the first gene-expression profile of *C. reinhardtii *under hydrogen-producing conditions, which was recently reported [102]. RNA-seq analysis of *C. reinhardtii *under nitrogen depleted conditions revealed statistically significant increases in several lipid biosynthesis genes including KASI, FAT1, and DGAT and decreases in beta oxidation genes such as LCS. Many of these genes were expected to be upregulated in lipid producing conditions, but more thorough bioinformatic analysis should yield new targets for genetic manipulation. More genomic, proteomic and metabolomic studies on algae lipid biosynthesis should also be nearing completion. The idea of algal oils as a potential biodiesel feedstock has been proposed and developed for years. The progresses in algal genetic engineering technology should accelerate any steps taken in achieving this goal.

## Concluding Remarks

Renewable energy has become an important issue of recent political campaigns, and an increase in usage with less reliance on fossil energy will create substantial benefits for the global environment, economy, and industry. Biofuels are one of the few renewable energies proposed that have generated large public expectation as a real possibility for one of the fuels of the future. The use and production of plant oil as a source of biodiesel is expanding annually. Decades of studies have provided a general scheme of the plant lipid metabolism, and genetic engineering methods have provided valuable data and several field trials. However, more studies in organism-scale metabolic regulation will be necessary to understand how plants control their lipid biosynthetic pathways in response to physiological and environmental conditions. Elucidation of complex flux-control will hold great benefits for future biofuel production.

Algae, the world's largest group of photosynthetic organisms, contribute a majority of the carbon fixation on earth, turning greenhouse gases into carbohydrates and lipids. Using algal oils as a biodiesel feedstock holds major advantages in comparison to plant oils. Algal cultures have long been studied, and already are used to produce several important value-added products for the agriculture and food industries, such as VLC-PUFA, carotenoids, and high-protein animal feeds. The carbohydrates and cellulosic cell wall of algae have the potential to be hydrolyzed and fermented into bioethanol, further increasing the utility of algae as a biofuel feedstock. Algae cells can also be used to synthesize important eukaryotic proteins or natural products for pharmaceutical applications. Further fundamental studies in algae metabolism hold the possibility of making the algae cell a multi-use feedstock and creating a true "green gold".

## Competing interests

The authors declare that they have no competing interests.

## Authors' contributions

All authors contributed to the background research and writing of the article, as well as the editing. In addition, all authors have read and approved the final version of this manuscript.
